# Neonatology Providers Need Education About Cystic Fibrosis Newborn Screening Algorithms

**DOI:** 10.3390/ijns11030054

**Published:** 2025-07-17

**Authors:** Nilesh Seshadri, Lori Christ, David Munson, Andrew Borowiec, Clement L. Ren, Ambika Shenoy

**Affiliations:** 1 Division of Pulmonary and Sleep Medicine, The Hub for Clinical Collaboration, Children’s Hospital of Philadelphia, 3500 Civic Center Blvd., Philadelphia, PA 19104, USA; seshadrin@chop.edu (N.S.); borowieca@chop.edu (A.B.); renc@chop.edu (C.L.R.); 2 Division of Neonatology, Children’s Hospital of Philadelphia, 3400 Civic Center Blvd., Philadelphia, PA 19104, USA; lori.christ@pennmedicine.upenn.edu (L.C.); munson@chop.edu (D.M.); 3 Perelman School of Medicine, University of Pennsylvania, Philadelphia, PA 19104, USA

**Keywords:** cystic fibrosis, newborn screening, neonatology, education

## Abstract

An essential link in the cystic fibrosis (CF) newborn screening (NBS) process is communication of results. While this is described between NBS programs and primary care providers, data of this occurrence is limited with neonatologists. Neonatology providers represent a group caring for critically ill infants with conditions that can impact their ability to complete diagnostic testing after an abnormal NBS. Delays in testing can prolong time to diagnosis. We fielded a survey to assess neonatology provider knowledge and awareness of the Pennsylvania state CF NBS algorithm after an update occurred. Provider demographics, awareness of CF NBS update, and knowledge of the diagnostic testing process were measured. 86% of respondents were unaware of Pennsylvania CF NBS updates. Provider comfort with interpreting CF NBS results varied. 40% of providers identified the next diagnostic testing steps for a critically ill infant following an abnormal CF NBS. Our survey emphasizes the need for educating neonatology providers about CF NBS to improve knowledge and awareness of CF NBS algorithms to facilitate the early diagnosis of affected infants.

## 1. Introduction

Newborn screening (NBS) is an essential tool for the early diagnosis of cystic fibrosis (CF) in neonates to optimize health outcomes [1]. CF NBS programs in the United States measure immunoreactive trypsinogen (IRT) levels from an infant’s dried blood specimen; if the IRT is elevated, DNA testing is performed to evaluate for cystic fibrosis transmembrane conductance regular protein (CFTR) variants [2]. Infants with abnormal CF NBS results require timely evaluation and sweat testing to diagnose CF [1]. Apart from test collection and analysis, additional key links to the CF NBS process are communication of test results with medical care providers and families to facilitate diagnostic testing to achieve rapid evaluation, identification, and treatment in affected infants to confer the benefits of early CF diagnosis [1].

Prior studies have evaluated the experiences of primary care providers in interpreting abnormal NBS results [3] and discussing positive CF NBS results with families [4]. However, there is limited data about neonatology provider awareness and understanding of CF NBS results. Neonatology providers represent an important group caring for infants, as infants under their care are medically complex and experience conditions that may impact NBS results or delay ability to complete diagnostic testing for CF [2]. For example, infants who are born prematurely or require parenteral feeds may have abnormal NBS results [5]. Furthermore, some infants hospitalized for managing meconium ileus may exhibit normal IRT levels and result in false negative NBS results despite having CF and requiring additional diagnostic evaluation [6]. The 2022 CF NBS algorithm update in Pennsylvania provided an opportunity to evaluate neonatology provider awareness and knowledge of the CF NBS process and subsequent diagnostic steps when required.

## 2. Materials and Methods

An educational needs survey was designed jointly by the Children’s Hospital of Philadelphia (CHOP) CF center and neonatology medical directors (see Supplemental Data). Survey questions assessed provider demographics, awareness of the state CF NBS update, and comfort with interpreting CF NBS results. Case-based clinical questions were utilized to evaluate understanding of requirements for sweat testing and knowledge of the Pennsylvania CF NBS algorithm in the Neonatal Intensive Care Unit (NICU) setting. The survey was distributed to neonatology providers from the level IV CHOP NICU and the level III Hospital of the University of Pennsylvania Intensive Care Nursery over a 4-week period in March 2024, approximately 18 months after the Pennsylvania NBS algorithm was updated. Survey responses were analyzed in aggregate anonymously. The majority of neonatology providers provided care at both NICU sites. This survey did not meet the CHOP IRB definition of human subjects’ research and was exempt from review.

## 3. Results

### 3.1. Neonatology Provider Demographics and Awareness of the Pennsylvania CF NBS Algorithm

There were 50/200 neonatology providers that completed the survey (25% response rate). Table 1 depicts neonatology provider demographics including provider type and years of practice. 86% (*n* = 43) of neonatology providers were unaware of the Pennsylvania CF NBS algorithm update.

### 3.2. Neonatology Provider Comfort with Interpreting Pennsylvania CF NBS Reports and Communicating Results to Families

**Figure 1:**
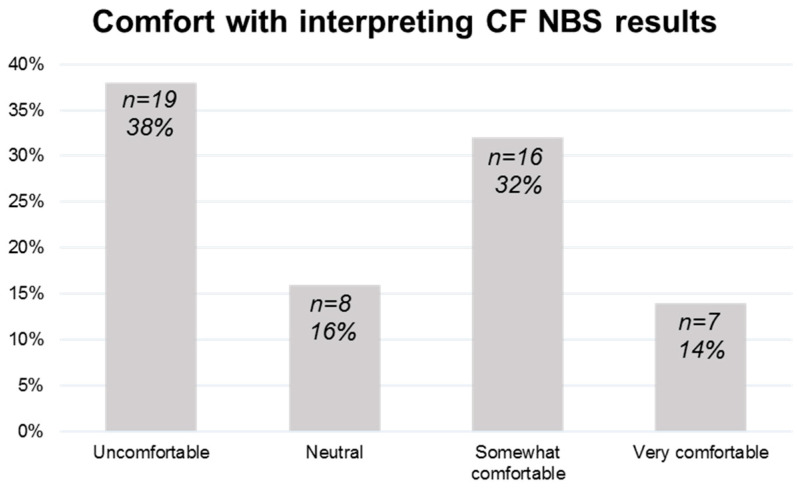
**Provider comfort with Pennsylvania Cystic Fibrosis (CF) Newborn Screening (NBS) results interpretation**. Comfort levels with interpreting state CF NBS reports varied. 46% of providers (*n* = 23) were comfortable or very comfortable with interpreting Pennsylvania CF NBS reports while 38% (*n* = 19) of providers were uncomfortable with the process.

Neonatology provider comfort with interpreting CF NBS reports varied. Responses ranged from 38% (*n* = 19) uncomfortable to 46% (*n* = 23) comfortable or very comfortable with interpreting Pennsylvania CF NBS results (Figure 1). Responses demonstrated that attending physicians (*n* = 45) and advanced practice providers (*n* = 33) were groups that most often discussed CF NBS results with families.

### 3.3. Neonatology Provider Knowledge of Diagnostic Evaluation Based on the Pennsylvania CF NBS Algorithm

**Figure 2:**
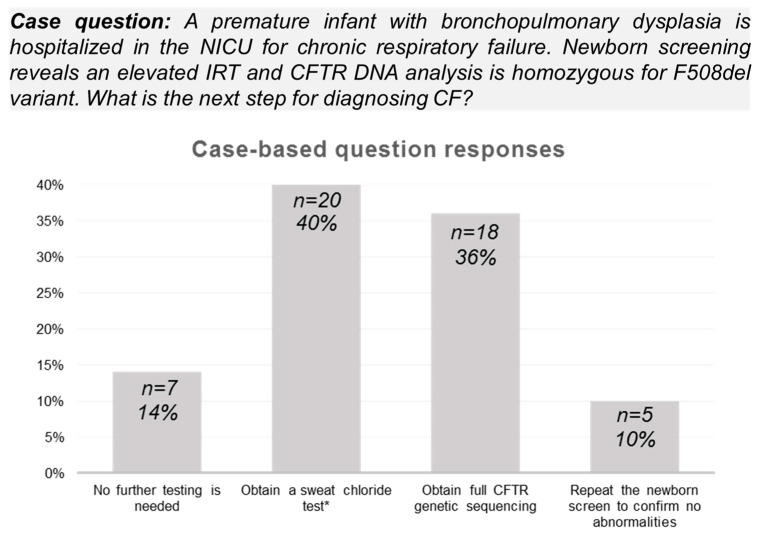
**Survey responses to case-based question about diagnostic evaluation following an abnormal Pennsylvania Cystic Fibrosis (CF) Newborn Screening (NBS) result.** Only 40% (*n* = 20/50) of respondents identified the need for confirmation of an abnormal CF NBS result (elevated immunoreactive trypsinogen (IRT) and homozygous F508del CFTR variant) with diagnostic testing (sweat chloride analysis) based on the state algorithm (correct answer denoted by an asterisk as shown).

Neonatology providers showed awareness of the minimum weight (64%, *n* = 32) and corrected gestational age (52%, *n* = 26) guideline requirements for successful sweat test completion. Less than half of neonatology providers (40%, *n* = 20) identified the next diagnostic testing steps based on the Pennsylvania CF NBS algorithm in an infant with an abnormal CF NBS result (Figure 2). Most providers (92%, *n* = 46) correctly identified the association between meconium ileus and cystic fibrosis. The majority of neonatology providers (98%, *n* = 49) used effective communication to review abnormal CF NBS results with families, introduce pulmonary consultation, and discuss the need for diagnostic testing.

## 4. Discussion

Recent CF Foundation guidelines for NBS have recognized the unique nature of critically ill neonates and cited recommendations for infants hospitalized in the NICU [2]. Infants in the NICU with abnormal CF NBS results represent a unique population since their medical status (e.g., prematurity, weight, and/or critical illness) may impact timing and ability to complete diagnostic testing successfully [7]. Neonatologists have identified the importance of screening for CF on the NBS panel [8]. However, to our knowledge, this is the first initiative that outlined neonatology provider knowledge and awareness of CF NBS protocols, particularly following an update to a state algorithm.

Infants who are diagnosed with CF in the neonatal period via NBS demonstrate improved health outcomes [9,10,11,12]. Therefore, the early diagnosis of CF within the first month of life is critical to achieving the long-term benefits of NBS. In this survey, neonatology providers were unaware of updates to the local Pennsylvania CF NBS algorithm. Thus, there is a need for neonatology providers to receive education about state and local CF NBS algorithms to facilitate identification and referral of infants with abnormal CF NBS results in a timely manner.

Neonatology providers surveyed were knowledgeable of CF diagnostic testing requirements and exhibited effective communication skills when discussing results with families. However, their comfort levels with interpreting CF NBS reports varied and further underscores the need for education about CF NBS processes by the state CF NBS program and local CF center. As the CF care team continues to evolve [13], additional partnerships between neonatology providers and outpatient CF practitioners are essential to facilitate prompt diagnosis and timely follow up, particularly for neonates with underlying medical complexity in the NICU setting.

Some limitations are noted in this project’s methodology and findings. First, our survey question regarding neonatology provider comfort in the interpretation of CF NBS results provided limited options to report the level of discomfort. As a result, a more detailed analysis into reasons for provider discomfort with CF NBS findings was not possible. Second, the survey was conducted with providers working in a level IV NICU, where hospitalized infants have higher needs and medical complexity. Thus, findings may be less applicable to neonatology providers in lower acuity settings. Nevertheless, this study shares important updates in neonatology provider awareness to changes in the Pennsylvania CF NBS algorithm and gaps in education that need to be met. Our project highlights the importance of increased neonatology and general provider awareness of testing protocols and result interpretation for the timely diagnosis of CF in critically ill infants.

Next steps in the improvement plan include the design and implementation of education resources and programming for neonatology providers based on survey feedback. Knowledge and comfort with CF NBS interpretation will be reassessed following such interventions to trend progress. It is anticipated that these results will raise awareness about CF NBS education for neonatology providers in other regions, especially as algorithms are updated to improve the early detection of CF.

## Figures and Tables

**Table 1:** Neonatology provider demographics and awareness of the Pennsylvania Cystic Fibrosis (CF) Newborn Screening (NBS)Algorithm.

	Number of Respondents (% of Responses)
**Provider type**	
Neonatologist	24 (48%)
Neonatology fellow	5 (10%)
Advanced practice practitioner	18 (36%)
Hospitalist physician	3 (6%)
**Years in practice**	
<1	1 (2%)
1–5	19 (38%)
6–10	11 (22%)
11–20	11 (22%)
>20	8 (16%)
**Neonatology provider awareness of CF NBS** **algorithm change**	
Yes	7 (14%)
No	43 (86%)

## Data Availability

Data is contained within the article or Supplementary Material.

## Supplementary Materials

The following supporting information can be downloaded at: https://www.mdpi.com/article/10.3390/ijns11030054/s1, Neonatology provider survey developed jointly by CHOP CF center and Neonatology leadership to assess neonatology provider awareness and knowledge of the Pennsylvania CF NBS algorithm and diagnostic evaluation following abnormal results.

## Abbreviations

The following abbreviations are used in this manuscript:

CF	cystic fibrosis
NBS	newborn screening
IRT	immunoreactive trypsinogen
CFTR	Cystic fibrosis transmembrane conductance regulator
CHOP	Children’s Hospital of Philadelphia
NICU	Neonatal Intensive Care Unit
