# Supplementary material for: Neonatology Providers Need Education About Cystic Fibrosis Newborn Screening Algorithms
Source: Int J Neonatal Screen. 2025 Jul 17;11(3):54. doi: 10.3390/ijns11030054 (PMC12286156; doi:10.3390/ijns11030054)
Supplement: Supplementary file 1 [file IJNS-11-00054-s001.zip › IJNS-3723275-supplementary.pdf]

CHOP Neonatology Cystic Fibrosis (CF) newborn screening needs assessment survey

1. Describe your role within the Division of Neonatology:
    a. Neonatologist
    b. Fellow physician
    c. Advanced practice provider (Nurse practitioner, Physician Assistant) specify: ____________________
    d. Hospitalist
    e. Nursing
    f. Other (please specify) ___________________

2. How many years of experience do you have providing neonatal care?
    a. <1 year
    b. 1-5 years
    c. 6-10 years
    d. 11-20 years
    e. >20 years

3. Are you aware of the recent changes in the Pennsylvania CF newborn screening algorithm to include expanded cystic fibrosis transmembrane regulator (CFTR) sequencing?
    a. Yes
    b. No

4. How comfortable are you interpreting results from a Pennsylvania CF newborn screening report?
    a. Very comfortable
    b. Somewhat comfortable
    c. Neutral
    d. Uncomfortable – need to look up more information or prefer to have subspecialty input
    e. Not applicable to my role

5. What is your approach to communicating abnormal CF newborn screening (NBS) results with families?
    a. Tell the family there are questions about NBS results, additional testing is needed, and the Pulmonology team will come speak to them.
    b. Tell the family there are abnormalities with the NBS results and the Pulmonology team will come speak to them and likely order additional testing.
    c. Tell family the results likely indicate CF and Pulmonology team will come speak to them.
    d. Call Pulmonology before discussing any results with the family.
6. Who is the most likely person to discuss abnormal CF newborn screen results with families?
    a. Attending physician
    b. Fellow physician
    c. Advanced Practice Provider (Nurse practitioner or Physician Assistant)

d. Hospitalist
e. Resident physician
f. Not standardized
g. Other (please specify) ________________________
h. I don't know

Case-based clinical questions:

Case 1: A 7-day old infant delivered at 39 weeks gestational age fails to pass stool within 48 hours. He was transferred to the neonatal intensive care unit due to concern for bowel obstruction on X-ray. Contrast enema suggests meconium ileus, and general surgery is consulted. The infant underwent bowel resection on DOL# 3 with an uncomplicated post-operative course. The infant does not have any respiratory symptoms, including cough or wheezing. They were successfully extubated post-procedure and are currently in room air. The current weight is 3.2 kg (birthweight 3.3 kg). His newborn screening is currently in process.

1. What is the minimum weight required for an infant to complete a sweat test?
   a. 1 kg
   b. 2 kg
   c. 5 kg
   d. No minimum weight

2. Which statement about meconium ileus and cystic fibrosis is TRUE?
   a. If a newborn screen is negative for CF, that is sufficient to exclude the diagnosis.
   b. Given the strong association with CF, all infants with meconium ileus should complete sweat testing.
   c. Sweat testing should only be performed in infants with meconium ileus if they develop chronic respiratory symptoms.
   d. There is no association between meconium ileus and cystic fibrosis.

Case 2: A 14-day old male infant is born at 28 weeks gestation and remains in the NICU due to chronic respiratory failure and feeding difficulties. At present, the infant requires continuous CPAP 6 cm H2O for respiratory support. They are receiving bolus feeds via a nasogastric tube. The present weight is 1.2 kg. There is no family history of cystic fibrosis. Newborn screening results demonstrated an elevated IRT of 80 ng/ml and CFTR DNA analysis identifies that the infant is homozygous for F508del variant.

3. Based on the current CF newborn screening algorithm, what is the next step for diagnosing cystic fibrosis?
   a. Repeat the newborn screen to ensure there are no abnormalities.
   b. Obtain a sweat chloride test.
   c. Obtain full CFTR gene sequencing.
   d. No further testing is needed

4. What is the minimum (corrected) gestational age at which a sweat test can be performed?
   a. 28 weeks
   b. 32 weeks
   c. 36 weeks

d. 40 weeks

5. The parents express concern about the results of the newborn screen. What is the best response in discussing these results with the family?
    a. The newborn screening test suggests cystic fibrosis, pulmonology will be consulted and additional testing will confirm the diagnosis.
    b. There is low suspicion for cystic fibrosis since neither parent has the condition.
    c. Because the IRT is under 100 ng/ml, this child does not meet criteria for cystic fibrosis.
    d. The baby will likely not be at any greater risk for respiratory infections in the future.

List any information that would be helpful for the CHOP CF Center to provide you in caring for critically ill infants with abnormal cystic fibrosis newborn screen results.
Free Text: ____
